# Expression of Hypoxia-Inducible Factor (HIF)-1a-Vascular Endothelial Growth Factor (VEGF)-Inhibitory Growth Factor (ING)-4- axis in sarcoidosis patients

**DOI:** 10.1186/1756-0500-5-654

**Published:** 2012-11-26

**Authors:** Argyris Tzouvelekis, Paschalis Ntolios, Andreas Karameris, Anastasios Koutsopoulos, Panagiotis Boglou, Andreas Koulelidis, Kostas Archontogeorgis, George Zacharis, Fotis Drakopanagiotakis, Paschalis Steiropoulos, Stavros Anevlavis, Vlassis Polychronopoulos, Dimitrios Mikroulis, Demosthenes Bouros

**Affiliations:** 1 Department of Pneumonology, University Hospital of Alexandroupolis, Medical school, Democritus University of Thrace, Thrace, Greece; 2 Pulmonary Biochemistry, Centre for Respiratory Research, University College London, London, UK; 3 Department of Pathology, Veterans Administration Hospital (N.I.M.T.S), Athens, Greece; 4 Department of Pathology, University Hospital of Alexandroupolis, Medical school, Democritus University of Thrace, Thrace, Greece; 5 Department of Pneumonology, Sismanogleio General Hospital, Athens, Greece; 6 Department of Cardiothoracic Surgery, University Hospital of Alexandroupolis, Medical School, Democritus University of Thrace, Thrace, Greece

## Abstract

**Background:**

Sarcoidosis is a granulomatous disorder of unknown etiology. The term of immunoangiostasis has been addressed by various studies as potentially involved in the disease pathogenesis. The aim of the study was to investigate the expression of the master regulator of angiogenesis hypoxia inducible factor (HIF)-1a – vascular endothelial growth factor (VEGF)- inhibitor of growth factor 4-(ING4) - axis within sarcoid granuloma.

**Methods:**

A total of 37 patients with sarcoidosis stages II-III were recruited in our study. Tissue microarray technology coupled with immunohistochemistry analysis were applied to video-assisted thoracoscopic surgery (VATS) lung biopsy samples collected from 37 sarcoidosis patients and 24 controls underwent surgery for benign lesions of the lung. Computerized image analysis was used to quantify immunohistochemistry results. qRT-PCR was used to assess HIF-1a and ING4 expression in 10 sarcoidosis mediastinal lymph node and 10 control lung samples.

**Results:**

HIF-1a and VEGF-ING4 expression, both in protein and mRNA level, was found to be downregulated and upregulated, respectively, in sarcoidosis samples compared to controls. Immunohistochemistry coupled with computerized image analysis revealed minimal expression of HIF-1a within sarcoid granulomas whereas an abundant staining of ING4 and VEGF in epithelioid cells was also visualized.

**Conclusions:**

Our data suggest an impairment of the HIF-1a – VEGF axis, potentialy arising by ING4 overexpression and ultimately resulting in angiostasis and monocyte recruitment within granulomas. The concept of immunoangiostasis as a possible protection mechanism against antigens of infectious origin needs further research to be verified.

## Background

Sarcoidosis is an immunologic, granulomatous disorder affecting multiple systems. It is pathologically characterized by the presence of non-caseating granulomas in involved organs [1]. Lung, including the mediastinal and hilar lymph nodes is the most common site of disease manifestation [2,3]. The prevalence of the disease is estimated at 10 to 20 per 100.000 population [2,3]. Its pathogenesis is unknown, although various factors including environmental and occupational exposures, infectious agents and genetic susceptibility have been implicated [4-6].

Various studies suggest that angiogenic and angiostatic factors contribute to the pathogenesis of Sarcoidosis [4,5,7-9]. Seminal observations by Strieter et al. [10] implicated angiogenesis in the pathogenesis of granulomatous and fibrotic lung disorders. A regulation of T cell migration and activation by angiostatic chemokines, such as IP-10, resulting in granulomas formation has also been demonstrated [10]. Further extending the latter observations, a distinct angiogenic and angiostatic profile between sarcoidosis and idiopathic pulmonary fibrosis (IPF) has been recently reported [8].

Vascular endothelial growth factor (VEGF) represents one of the most potent mediators of angiogenesis both in vivo and in vitro. Beyond that, VEGF presents with major pleiotropic properties. It has been identified to regulate monocyte recruitment towards granuloma formation, while its expression within sarcoid granuloma through the *flt* receptor was perceived to be elevated [11]. Nevertheless its exact role in disease pathogenesis is still elusive and controversial [12]. Genetic polymorphisms of VEGF have been associated with disease susceptibility and may explain discrepancies in VEGF levels in sarcoidosis patients [11,13]. VEGF expression is mediated by hypoxia inducible factor (HIF)-1a [14]. HIF-1a is recognized as a master regulator of hypoxic signaling by activating gene transcription of genes encoding proteins mediating the cellular adaptive response under hypoxic conditions [14-17]. Nevertheless, an inflammatory microenvironment can trigger HIF-1a expression even under normoxic conditions [18]. Our group implicated for the first time HIF-1a in the pathogenesis of IPF. We demonstrated an overexpression of HIF-1a and its transcription genes involved in angiogenesis (VEGF) and apoptosis (p53) mainly localized within alveolar epithelium of the fibrotic lungs [19]. Further extending our seminal observations, we have recently reported a downregulation of inhibitor of growth factor (ING)-4 in IPF lung samples. ING4 is a potent suppressor of HIF-1a that exerts a beneficial role in cancer invasion, migration and metastasis by inhibiting cell proliferation and angiogenesis [20-23].

Our recent observations triggered the idea that the concept of "immunoangiostasis" could provide a reasonable explanation for sarcoid granuloma formation. Immunoangiostasis concept supports that an angiostatic and avascular microenvironment may preserve the responsible infectious agent under dormant state, while at the same time will facilitate its eradication by monocyte recruitment [24,25]. We therefore utilized high-throughput microarray technology and computerized image analysis seeking to determine the expression of HIF-1a-VEGF-ING4 axis in lung biopsy samples from patients with sarcoidosis of stages II-III.

## Patients and methods

### Patients

A total of 37 patients with pulmonary sarcoidosis were recruited in the study (Table 1). Diagnosis of sarcoidosis was based the following criteria: 1) compatible clinical and radiological picture, 2) the histological evidence of non-caseating granulomas and 3) exclusion of other diseases capable of producing a similar histological or clinical picture [3]. Approval of the Ethics Committee of the Democritus University of Thrace, Greece was obtained (reference number 1669/2010). Part of the video assisted thoracoscopic (VATS) lung biopsy tissue was used to establish a diagnosis and the rest were formalin fixed and paraffin embedded to be used for tissue microarray construction. Twenty nine patients were of radiological stage II, 22 were male and 17 were never-smokers. Based on functional assessment patients had mild-to moderate restrictive pattern with a mean forced vital capacity (FVC): 72 ± 7%pred, forced expiratory volume at 1s (FEV1): 77 ± 11%pred, an FEV1/FVC: 85 ± 13, a total lung capacity (TLC): 64 ± 10%pred, and a moderate gas transfer impairment as estimated by diffusing lung capacity for carbon monoxide (DL_CO_): 69 ± 9%pred. Twenty four control paraffin blocks obtained from the normal part of lungs removed for benign lesions were collected from the archives of the Department of Pathology of Veterans Hospital, Athens, Greece.

**Table 1:** Baseline and functional characteristics of the study population

**Gender**	
Female	15
Male	22
**Mean age, years (range)**	54 (34–72)
**Radiological stage**	
II	29
III	8
**Smoking history**	
Current smokers	0
Ex smokers	20
Never smokers	17
**Functional data**	
FEV_1_%pred	77 ± 11
FVC %pred	72 ± 7
FEV_1_/FVC	85 ± 13
TLC %pred	64 ± 10
DL_CO_ %pred	69 ± 9
**Treatment (n)**	
*Corticosteroids*	37
*Azathioprine*	8

Data are presented as median (range), No (total) or mean ± SD, unless stated otherwise.

Abbreviations: DL_CO_: Diffuse lung capacity for carbon monoxide, FEV_1_: Forced Expiratory Volume in one second, FVC: Forced vital capacity, TLC: Total lung capacity

## Methods

### Quantitative Real-Time reverse transcriptase-polymerase chain reaction (qRT-PCR)

qRT-PCR was performed using the Chromo 4 Real-Time Detection System and the Platinum® SYBR® Green qPCR SuperMix-UDG (Invitrogen) on 10 sarcoidosis lung tissue samples and 10 control lung samples obtained during surgical removal of benign lesions of the lung, according to the manufacturer's instructions. The program used included: 2 min at 50°C, 5 min at 95°C, 43 cycles of denaturation-annealing- extension (30s at 95°C; 45s at 56°C; 30s at 72°C) and a final extension of 5 min at 72°C. Primers were chosen from exons separated by large introns (spanning exonexon junctions), and the PCR quality and specificity was verified by melting curve analysis and gel electrophoresis. Human (h) primer sequences (s: sense, as: antisense) and expected lengths (in bp) were as follows (5' to 3'): ING4 (s: AGC TTG CCA TGC AGA CCT; as: GCG CAC GAG CTT TAA CTT; 245 bp) B2M (s: CTG ACC CTA CAT TTT GTG CAT AAA AGATG AGT ATG CC; as: ACC CTA CAT TTT GTG CAT AA; 202 bp). HIF-1A (s: GAA AGC GCA AGT CTT CAA AG; as: TGG GTA GGA GAT GGA GAT GC; bp). VEGF (s: CTA CCT CCA CCA TGC CAA GT; bp) Cycle threshold (Ct; the first cycle that amplification can be detected) values were obtained from the Opticon monitor 3 software for each gene of interest and the control reference gene, together with amplification efficiencies (85–115%). Ct values were normalized to the reference gene beta-2-microglobulin (B2M) [14].

### Tissue microarrays

A total of 61 lung tissue samples including 37 sarcoidosis and 24 control tissues extracted from the normal part of the lung removed for benign lesions were snap frozen and stored at 70°C. Specimens were fixed in cold-ethanol for 16 h and then embedded in paraffin. Hematoxylin and eosin (H&E) –stained slides were used from each block to define the regions characterized by granulomatous lesions. Areas of interest were verified in H&E stained slides by using a conventional microscope (Olympus BX-50). Tissue cylinders of 1.5 mm diameter were punched from selected areas of each "donor" block by utilizing a thin-wall stainless tube from a precision instrument (TMA-100, Chemicon, USA) and were transferred by a solid stainless stylet into defined array coordinates in a 45 * 20 mm new recipient paraffin block (citation 17 from ING paper). The tissue microarray blocks were constructed in three copies (each containing one sample from a different region of all lesions). One sample was taken from the centre and two samples from different peripheral areas. Ultimately, we created two tissue microarray blocks comprising of 60 tissue elements each. Each tissue element in the array was 1.5 mm in diameter and spacing between two adjacent elements was 0.1 mm. After the tissue microarray construction 3 μm sections for immunohistochemical analysis were cut from the "donor" blocks and were transferred to glass slides using an adhesive-coated tap sectioning system.

### Immunohistochemistry

Immunohistochemistry was performed by using specific monoclonal antibodies for HIF-1a (HIF-1a-rabbit anti-human antibody, Abcam Lim), VEGF for 121, 165 and 189 isoforms, and ING4 (anti-h-ING4 rabbit polyclonal unconjugated antibody (10617-1-AP-Proteintech Group, Inc., Chicago, IL, USA). The slides were deparaffinised and En Vision immunohistochemistry protocol (DAKI corp, Denmark) was carried through the use of an automated immunohistochemistry staining system (Bond-Biogenex, USA). Diaminobenzidine (DAB) was used as chromogenic substrate. This immunohistochemistry protocol is based on a water-soluble, dextran polymer system preventing the endogenous biotin reaction, which is responsible for the background in the stained slides. More specifically, the sections were incubated with the primary antibody in "antibody diluent" (DAKO) and goat-anti-mouse EnVision- HRP-enzyme conjugate was performed for 3 min each. The "highly sensitive 3,3,' diaminobenzidine plus" (DAB+) and the "3- amino-9-ethylcarbazol plus" (AEC+) chromogens (both from DAKO) were used as substrates for the EnVision- HRP-enzymes. Staining intensity was further enhanced by modifying the manufacturer's protocol in that all incubation steps (primary antibodies, EnVision, and substrate reactions) were performed on slides placed horizontally on a thermal plate at 37C. After each incubation the slides were dipped in TBS or, after the substrate reaction, in tap water at RT and waved at maximum speed for 10 sec. Excess liquid (buffer/water) was soaked up by a paper towel. Specimens of colon adenocarcinoma cases were used as positive controls for the marker.

### Computerized image analysis

In order to evaluate the immunohistochemistry results not in a qualitative way but in a more accurate and reliable way, we performed computerized image analysis, in all tissue microarray samples (n=61), by using a semi-automated system (Matrox II Card Frame Grabber, Camera Microwave Systems, Microscope Olympus BX-50) allowing us to assess staining intensity in a 256 level scale – 0 (black)-255(white). Staining intensity values were then converted to reverse percentages [reverse staining intensity = (1-staining intensity/256) ×100].

### Statistical analysis

Statistical analysis was carried out using SPSS 14.0 software. Results are expressed as mean ± SD, or median (range), unless otherwise indicated. Statistical significance was verified by performing independent samples *t*-test to compare reverse staining intensity and qRT-PCR values of HIF-1a and VEGF-ING4 between patients and controls. Results were corrected using Bonferroni correction. A p-value of < 0.05 was considered as statistically significant.

## Results

### Absence of HIF-1a expression within sarcoid granulomas

**Figure 1:**
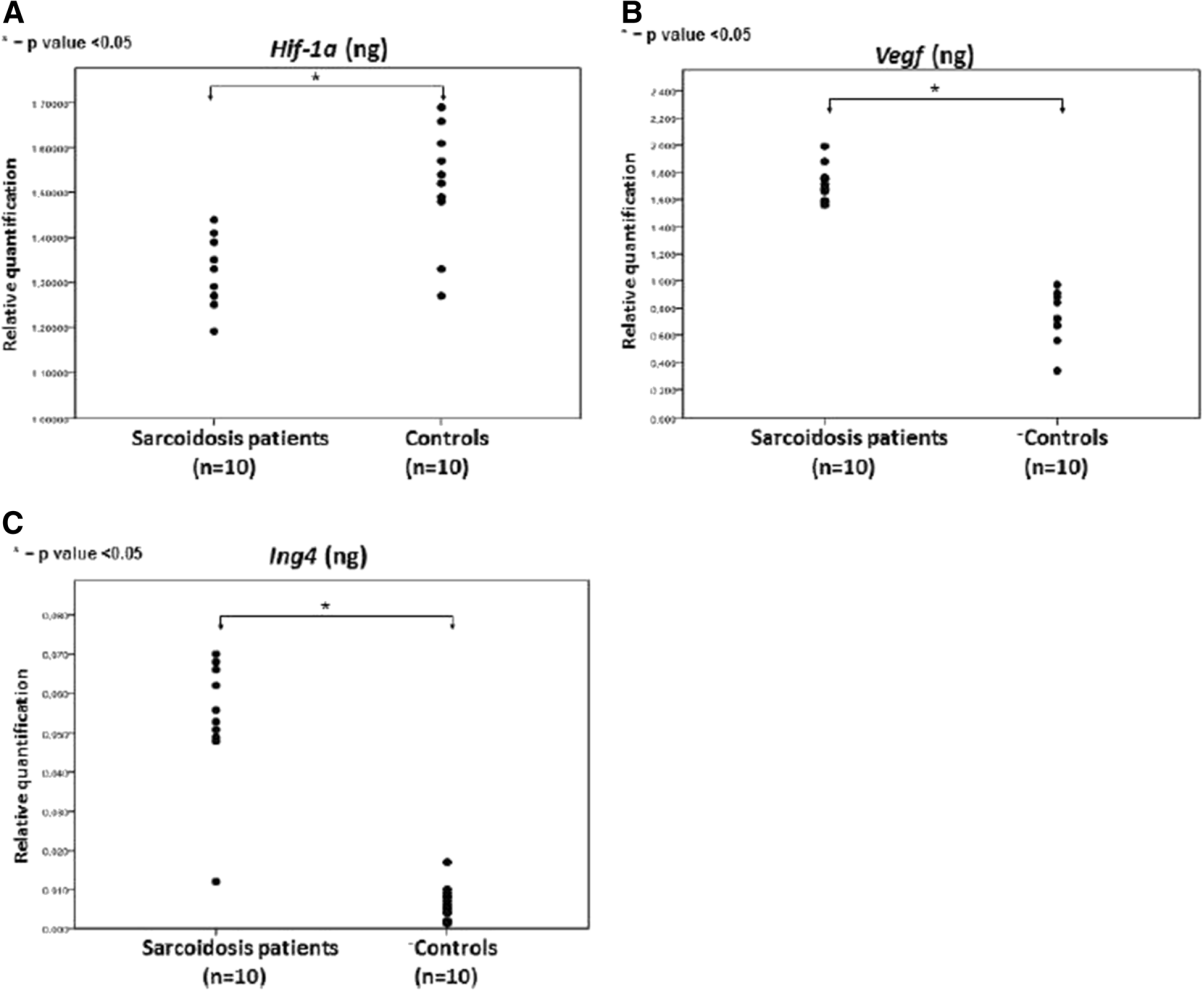
***HIF-1a, Vegf and Ing4 *mRNA expression levels in sarcoidosis lung samples.*** Hif-1a* (**A**)*, Vegf* (**B**) and *Ing4* (**C**) gene expression levels quantified by qRT-PCR showed a statistically significant incline and decline in sarcoidosis lung tissue samples (n=10) compared to control lung samples (n=10). All values were normalized with the reference gene *B2m*. *p < 0.05.

**Figure 2:**
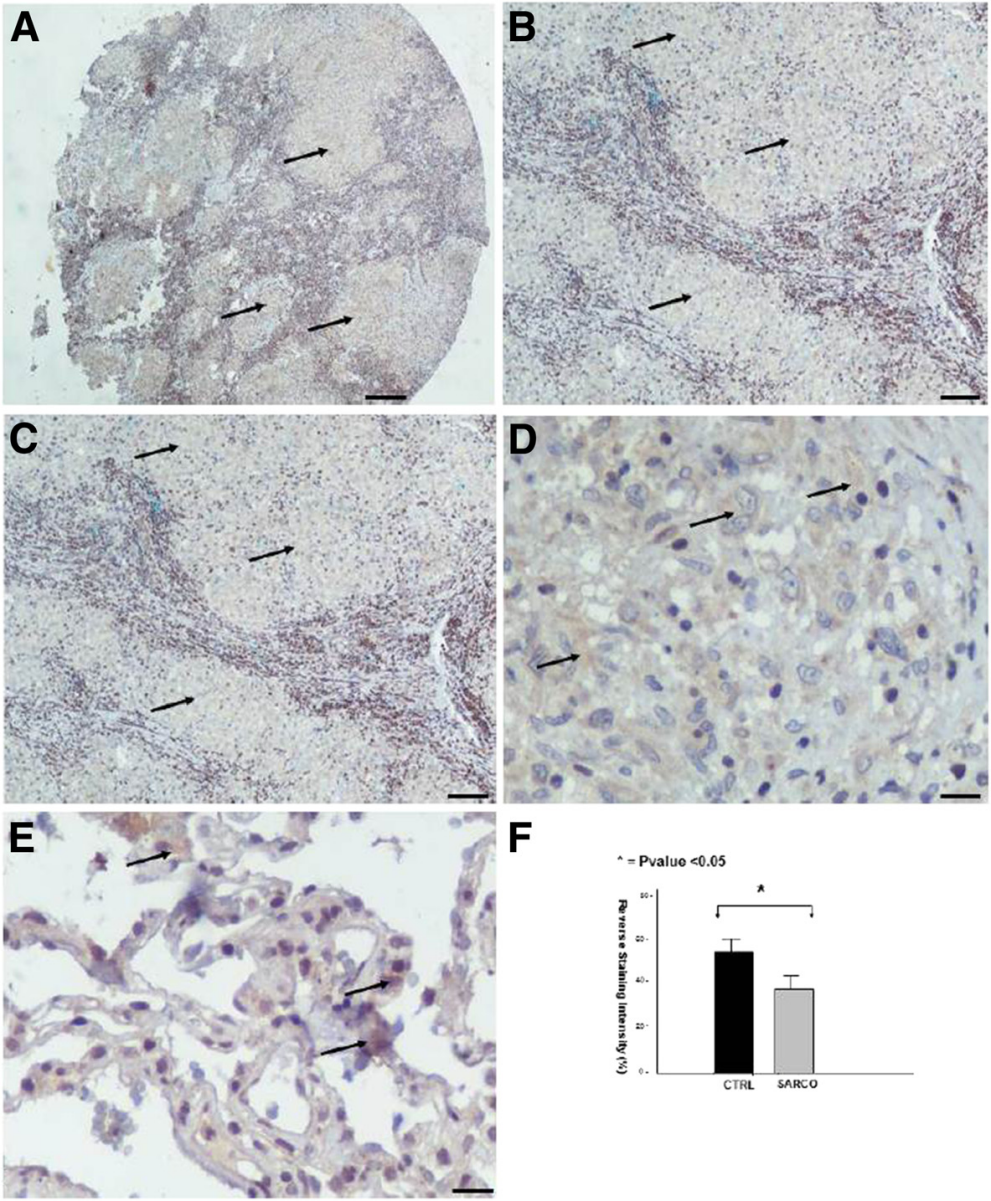
**HIF-1a immunohistochemical staining in sarcoidosis lung samples.** Representative tissue microarray section immunostained with monoclonal antibody HIF-1a demonstrating diffuse cytoplasmic reaction of weak intensity in epithelioid cells within granulomas (arrows) derived from sarcoidosis patients (n=37) compared to control lung samples (n=24) (E). Scale bars in panel **A**: 100μm, **B** and **C**: 25 μm and **D**, **E**: 10 μm. Immunohistochemical findings were confirmed by computerized image analysis (**F**).

HIF-1a was found to be minimally expressed within sarcoid granulomas when compared to control lung samples. Specifically, qRT-PCR in 10 sarcoidosis lung tissue samples revealed a downregulation of HIF-1a expression both in protein and mRNA level, compared to 10 controls, as shown in Figure 1A. To authenticate the above procedure, tissue microarray sections were stained with monoclonal HIF-1a antibody and demonstrated diffuse cytoplasmic reaction of weak intensity within granuloma (Figure 2A-D) compared to control specimens (Figure 2E). Results were further corroborated and quantified by computerized image analysis (Figure 2F)

### Abudant VEGF expression within sarcoid granulomas

**Figure 3:**
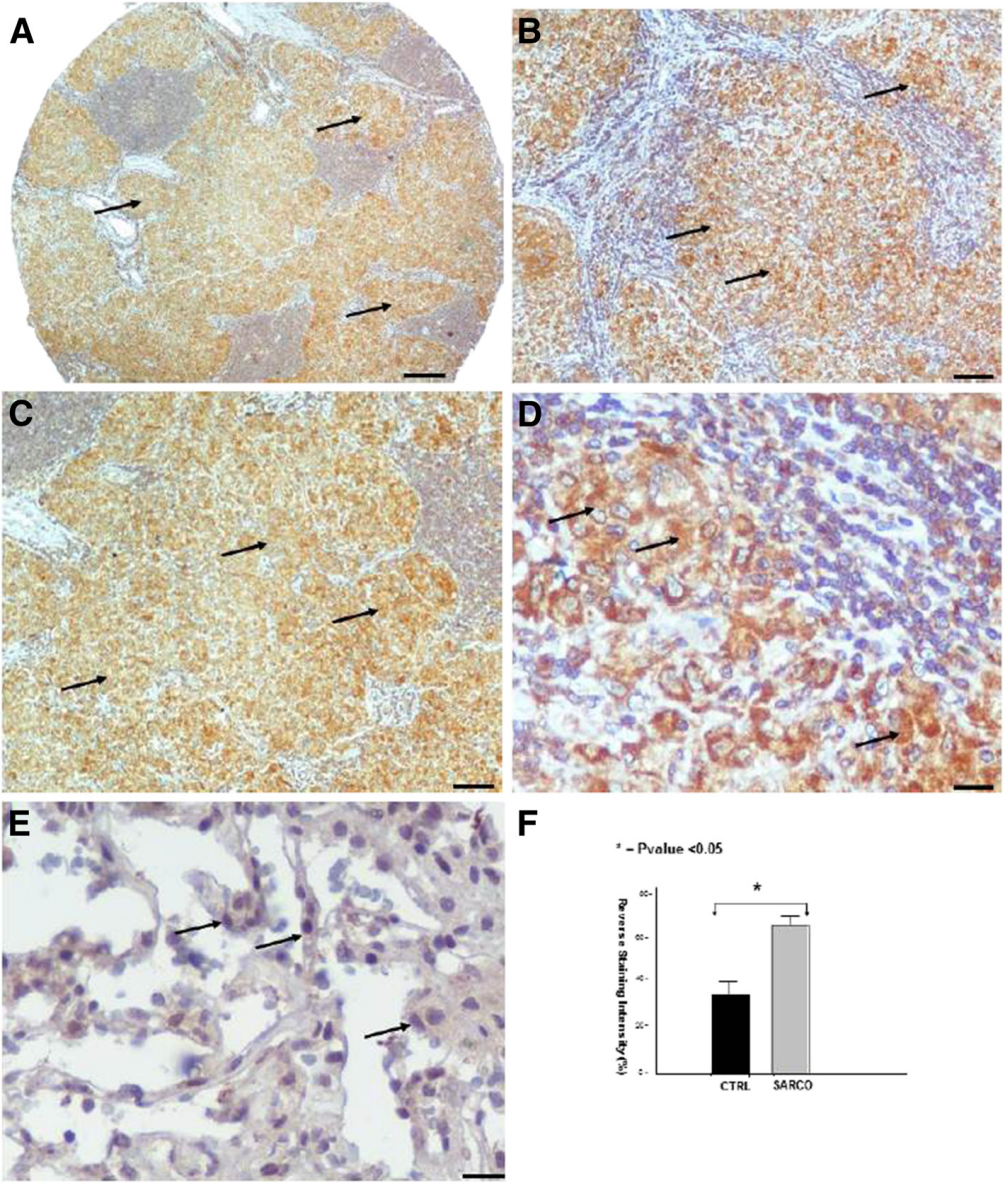
**VEGF immunohistochemical staining in sarcoidosis lung samples.** Representative immunohistochemically stained tissue microarray section with monoclonal antibody against VEGF demonstrates diffuse cytoplasmic stain of moderately strong intensity in epithelioid cells within granulomas (arrows) derived from sarcoidosis patients (n=37) compared to control lung samples (n=24). Scale bars in panel **A**: 100μm, **B** and **C**: 25 μm and **D**, **E**: 10 μm. Immunohistochemical findings were confirmed by computerized image analysis (**F**).

VEGF expression profile followed that of its master regulator, namely HIF-1a. In particular, qRT-PCR in 10 sarcoidosis and 10 control lung samples revealed abundant VEGF expression (Figure 1B) in sarcoidosis mediastinal lymph nodes compared to controls evidence that was further corroborated by semi-quantitative computerized immunohistochemistry analysis in tissue microarray sections of sarcoidosis patients revealing a diffuse cytoplasmic strong staining intensity, indicating increased VEGF expression (Figure 3A-F).

### Increased ING4 expression within sarcoid granulomas

**Figure 4:**
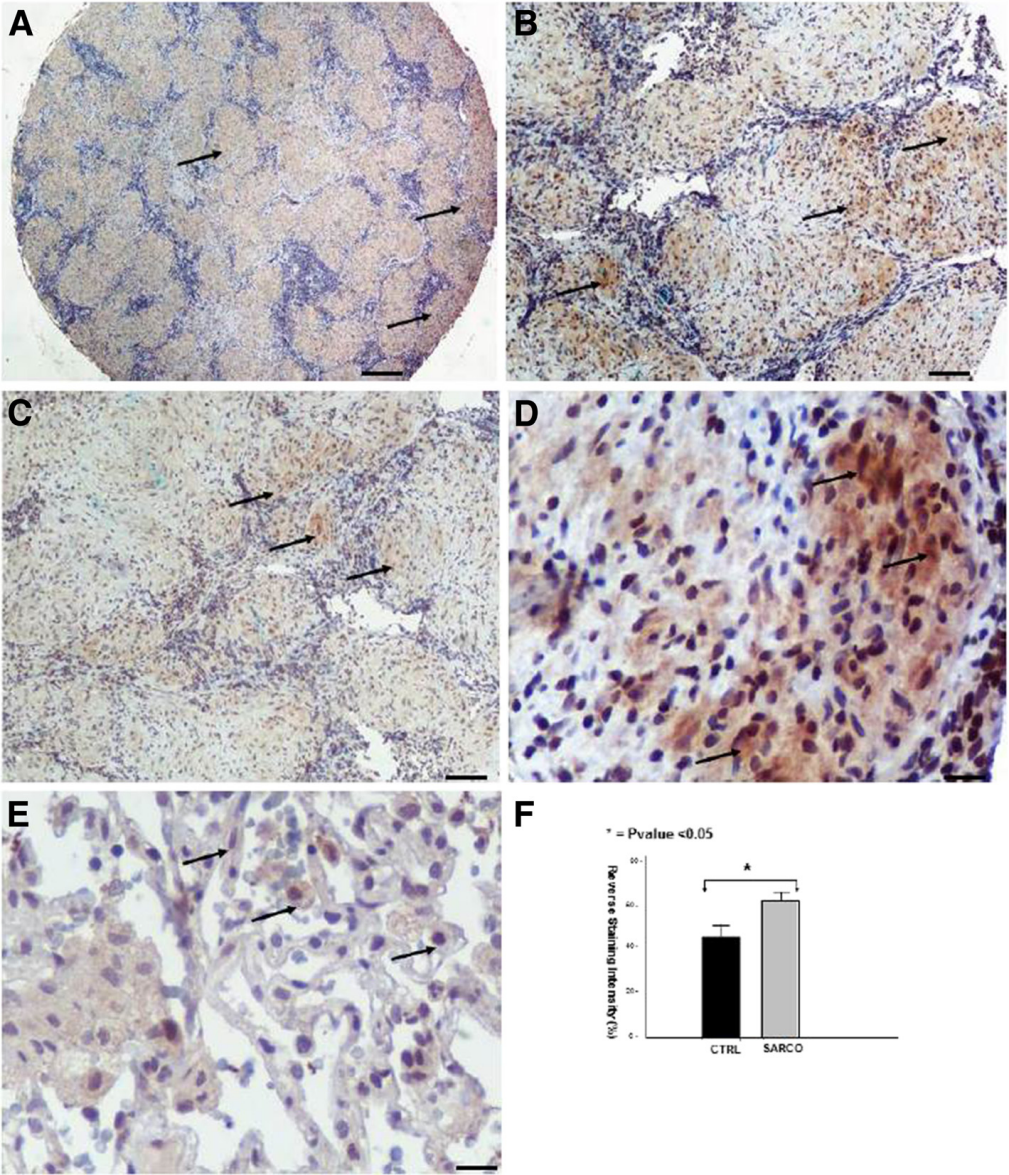
**ING4 immunohistochemical staining in sarcoidosis lung samples.** Representative immunohistochemical staining with monoclonal antibody against ING4 shows positively stained epithelioid cells within within granulomas (arrows) derived from sarcoidosis patients (n=37) compared to control lung samples (n=24). Scale bars in panel **A**: 100μm, **B** and **C**: 25 μm and **D**, **E**: 10 μm. Immunohistochemical findings were confirmed by computerized image analysis (**F**).

The expression pattern of ING4 within fibrotic lungs [20] was inversely related with that of HIF-1a, as has previously been demonstrated [19], suggesting a role for this transcription factor during disease pathogenesis. In line with the above existing literature, ING4 protein and mRNA levels were extensively expressed in areas of diminished HIF-1a expression, as assessed by qRT-PCR (Figure 1C), and tissue microarray immunohistochemical computerized image analysis, respectively. The latter starkly demonstrated positively stained epithelioid cells within sarcoid tissue samples with patchy distribution and strong intensity compared to control lung samples(Figure 4A-F).

## Discussion

To the best of our knowledge this is the first study in the literature investigating the expression of HIF-1a-VEGF-ING4- axis in sarcoidosis patients. High-throughput tissue microarray technology coupled with qRT-PCR, immunohistochemistry and computerized image analysis were applied to lung and lymph nodes biopsy samples from sarcoidosis patients and controls. Our results revealed a diminished expression, both in protein and mRNA level of HIF-1a within sarcoid granulomas and abundant expression of VEGF and ING4, mainly localized in epithelioid cells within granulomatous tissue.

HIF-1a expression represents the first step of cellular response to hypoxia, leading to the transcription of several genes involved in angiogenesis, apoptosis, cellular differentiation and proliferation as well as energy metabolism [14-16]. Until recently, its role in the pathogenesis of fibroproliferative lung disorders, including IPF has been severely underscored by the lack of sufficient evidence to support its role in fibrogenesis. Our study group seminally reported an early overexpression of HIF-1a during disease course both in the experimental model of pulmonary fibrosis and in actual human disease. Thus, suggesting that HIF-1a activation can represent an early event and a potential fibrotic stimulus [19]. HIF-1a activation can also be triggered by several inflammatory and immunomodulatory factors including insulin growth factor (IGF) and tumor necrosis factor (TNFa) [14,16,18]. Its expression is tightly regulated not only by changes in cellular O_2_ concentration [26] but can be inhibited by several growth factors including ING4 [23,27]. The latter seems to inhibit angiogenesis through interaction with HIF proly hydroxylases (HPH) and RelA subunit of NF-κΒ resulting in downregulation of *HIF* activation and repression of angiogenesis related genes including *IL6, IL-8 and Cox2*, respectively [23,27].

Sarcoidosis represents a disease paradigm with a predominance of the Th1 response in its immunopathogenesis [6]. The past years have seen the emergence of Th1 mediators such as CXCR3/CXCR3 ligands (interferon gamma regulated CXC chemokines) as factors leading to granuloma formation, the pathogenetic hallmark of sarcoidosis. These mediators have pleiotropic properties including inhibition of angiogenesis, monocyte recruitment and T cell migration to sites of ongoing inflammation [5-7,25]. In addition our study group demonstrated a distinct angiogenic and angiostatic profile between patients with IPF and sarcoidosis, further supporting the role of angiogenesis and its mediators in chronic lung disorders [8].

Despite these data there is pivotal lack of knowledge regarding the expression and the immunolocalization of the master regulator of angiogenesis HIF-1a and its related genes within the sarcoid granuloma. Therefore we applied the pioneering technology of tissue microarrays, which allowed us the simultaneous analysis of up to 61 lung samples from sarcoidosis patients and controls in a single experiment under highly standardized conditions [28]. Thus, all tissue samples were analyzed in an identical, unbiased fashion, with minimal tissue damage and precise positioning of arrayed samples, which served as an ideal basis for computerized image analysis of immunohistochemistry findings amenable to robust statistics. We identified a diminished expression of HIF-1a both in protein and mRNA level within sarcoid granuloma whereas an abundant expression of VEGF by epithelioid and giant cells in was also noted within granulomatous tissue. Furthermore to gain a holistic view regarding the hypoxia-angiogenesis axis within granulomatous lesions in sarcoidosis patients we investigated the expression of HIF-1a repressor gene, ING4. Immunohistochemistry and computerized image analysis revealed a strong staining intensity of ING4 in epithelioid cells within sarcoid granuloma, evidence that was further corroborated by elevated levels of mRNA, as assessed by qRT-PCR.

The impairment of the HIF-1a-VEGF axis may have dual explanation. Current data support the notion of an avascular micro-environment within sarcoid lesions [29]. Thus, it is conceivable to speculate that VEGF abundant expression may be implicated more in the inflammatory cascade of sarcoidosis rather than the angiogenic one. This is supported by the pleiotropic properties of VEGF to promote Th1-dependent immunity through facilitation of monocyte recruitment and T-cell migration to sites of ongoing inflammation [11,30].

An alternative explanation is that HIF-1a depreciated expression maybe attributed to the specific timepoint when the biopsy was taken during the disease course. A significant limitation of pathology studies is that the presented findings simply represent a snapshot of disease pathogenesis and by no means do they mirror the entire pathogenetic cascade. It is therefore impossible to conclude a causal-effect relationship between decline of HIF-1a expression and upregulation of VEGF and ING4 since HIF-1a may have been simply consumed and exerted its functions by the time diagnosis of sarcoidosis was established based on tissue findings. In line with this, based on the descriptive nature of our study, we cannot draw definite conclusions with regards to HIF-1a and ING4 contribution to immunoangiostasis. HIF-1a promotes angiogenesis through the transcription of various mediators, including VEGF which was found to be abundantly expressed inside granulomas. However, this notion deserves further investigation, since HIF-1a initiates a cascade of angiogenesis involving the activation of several transcriptional genes beyond VEGF.

## Conclusions

In sum, our data exhibit for the first time in literature a downregulation of HIF-1a within sarcoid granulomas, coupled with VEGF and ING4 upregulation. Reduced activity of HIF-1a due to ING4 overexpression may promote an angiostatic environment within granulomas, while VEGF could be responsible for macrophage chemotaxis. Our results represent the first attempt in the literature investigating the expression and immunolocalization of HIF-1a-VEGF-ING4 axis within sarcoid lesions shedding further light in the understanding of the pathogenesis of sarcoidosis, favoring the infectious etiology concept. More studies exploring the contribution of immunoangiostasis to the development of granulomas are urgently needed.

## Abbreviations

BALF: Bronchoalveolar Lavage fluid; DL_CO_: Diffuse lung capacity for carbon monoxide; FEV_1_: Forced Expiratory Volume in one second; FVC: Forced vital capacity; HIF-1a: Hypoxia Inducible Factor-1a; IPF: Idiopathic Pulmonary Fibrosis; ING4: Inhibitor of Growth Family member 4; MHC: Major Histocompatibility Complex; qRT-PCR: quantitative Real Time Polymerase Chain Reaction; TLC: Total lung capacity; VATS: Video-assisted Thoracic Surgery; VEGF: Vascular Endothelial Growth Factor.

## Competing interests

AT is a recipient of an unrestricted grant provided by Hellenic Thoracic Society for the years 2010–2012. PN is a recipient of a European Respiratory Society Long-Term Fellowship for the years 2011–2012.

## Authors’ contributions

AT and DB were involved with the study conception. AT, PN, AK, KA, GZ, FD, PS, SA and VP recruited the patients in the study. AT performed the statistical analysis of the manuscript. AT carried out the semi-quantitative immunohistochemical computerized image analysis of the tissue sections. AT and AK constructed the tissue microarrays. AK and AK set the histological diagnosis of sarcoidosis. AK provided us with the controls tissue samples. DM performed the VATS lung biopsies. AT performed the qRT-PCR. AT and PN prepared the manuscript. DB, VP and DM were involved in revising the article for important intellectual content. All authors read and approved the final manuscript.
